# Rapid Quantification of Riboflavin in Milk by Front-Face Fluorescence Spectroscopy: A Preliminary Study

**DOI:** 10.3390/foods9010006

**Published:** 2019-12-20

**Authors:** Ulises Alvarado, Anna Zamora, Jinfang Liu, Jordi Saldo, Manuel Castillo

**Affiliations:** 1 Centre d’Innovació, Recerca i Transferència en Tecnologia dels Aliments (CIRTTA), Departament de Ciència Animal i dels Aliments, Facultat de Veterinària, Universitat Autònoma de Barcelona, 08193 Bellaterra, Spain; ulises_alvarado@hotmail.com (U.A.); anna.zamora@uab.cat (A.Z.); jinfang.liu@cau.edu.cn (J.L.); jordi.saldo@uab.cat (J.S.); 2 Escuela Profesional de Ingeniería Agroindustrial, Facultad de Ciencias Agrarias, Universidad Nacional del Altiplano, Av. Floral 1153, Puno 21001, Peru

**Keywords:** front-face fluorescence, milk, riboflavin, prediction, rapid quantification

## Abstract

The front-face fluorescence spectroscopy technique was used to establish a rapid prediction model of riboflavin concentration in milk without prior sample preparation. The prediction model developed was then compared with two conventional high performance liquid chromatography (HPLC)-based quantification methods. The method of standard addition allowed detecting a linear correlation between fluorescence intensity and riboflavin concentration in 12% (*w*/*w*) reconstituted low-heat milk powder. Validation of the model yielded an R^2^ of 0.99 with a standard error of prediction of 0.13 mg/L. The results suggest a potential use of front-face fluorescence spectroscopy as a simple method for off- and in-line determinations of riboflavin in milk.

## 1. Introduction

Several methods have been developed for the identification and quantification of riboflavin in milk. Among them are HPLC with fluorescence detector (HPLC-FLD) [1,2], ultra violet-visible HPLC (HPLC-UV-Vis) [3], and spectrofluorimetric [4] methods. The disadvantages of these analytical procedures are that they all require complex sample preparation, extraction, and cleaning; they also need sophisticated measuring equipment, expensive reagents, qualified personnel, and a long time of analysis.

Front-face fluorescence spectroscopy (FFF) shows great potential for the development of rapid, non-destructive analytical techniques with high sensitivity and specificity for the identification and characterization of different micronutrients and analytes, directly in food [5]. Ayala et al. [6] obtained prediction models of lactulose concentration with one, two, and three variables, using as predictors the fluorescence of tryptophan, dityrosine, and Maillard compounds. In parallel, an optimum prediction model for retinol concentration in milk after heat treatment was obtained by Liu et al. [7] using three fluorescent predictors (tryptophan, Maillard compounds, and riboflavin).

Riboflavin is a water-soluble vitamin that exhibits native fluorescence, a property that could allow monitoring the changes associated with processing of foods containing it, facilitating not only the use of FFF for rapid quantification of riboflavin but also its use as a quick marker for the development of in-line process control sensors. In fact, Miquel Becker et al. [8] demonstrated that FFF spectroscopy could be used to monitor riboflavin content in yogurt. In this study, the correlation of obtained fluorescence landscapes with excitation wavelengths from 270 to 550 nm, emission wavelengths in the range 310 to 590 nm, and riboflavin content determined by the standard Association of Official Agricultural Chemists (AOAC) fluorometric method [4] was evaluated. The obtained model using a partial least square regression showed a high correlation (*R* = 0.99) and a prediction error of 0.092 μg riboflavin/g. However, there are no studies on the potential of FFF for the analysis of riboflavin content in milk. This methodology would enable the simple use of fluorescence detection systems, which would not require highly qualified personnel and would allow expressing the amount of riboflavin in real time in an economically affordable manner. Therefore, the objective of this work was to evaluate the feasibility of a quick quantification methodology of riboflavin in milk by FFF without the need of any sample manipulation and through a simple mathematical model, i.e., without chemometrics. Feasibility was evaluated by calibration and validation of a prediction model developed using independent data sets. It should be emphasized that the formal validation of the analytical method was not the subject of the present preliminary study.

## 2. Materials and Methods

The development of the proposed methodology consisted of two stages. In the first stage, a prediction model for quick determination of riboflavin concentration was developed and validated. In the second stage, the developed mathematical model was validated with commercial milk samples of several brands.

### 2.1. Development of a Prediction Model

A set of calibration samples was prepared by increasing the addition of riboflavin (0.0, 1.0, 1.5, 2.0, 2.5, and 3.0 ppm) in 12% (*w*/*w*) reconstituted “low heat” skim milk with an initial riboflavin content of 1.824 ppm quantified by the HPLC method of Albala-Hurtado et al. [3]. Riboflavin used was supplied by Sigma Aldrich (R9504, Sigma Aldrich, Saint Louis, Missouri). The range of concentrations used was selected on the basis of the usual contents of riboflavin in milk reported in the literature [9,10]. Three independent replicates of each concentration were prepared.

FFF determinations were performed using a Cary Eclipse Fluorescence Spectrophotometer (Agilent Technologies, Madrid, Spain) equipped with 15 W “press Xenon lamp” and a “front-face” geometry accessory (Solid Sample Holder accessory and cuvette Kit, Agilent Technologies) adjusted to an angle of incidence of 30°, which minimizes both specular phenomena from the surface of the cuvette and the inner filter effect. Measurements were made at 20 °C using Suprasil^®^ quartz cuvettes (UV fluorescence cell, Agilent Technologies, Madrid, Spain). In the present study, the excitation and emission wavelengths were set at 370 nm and 530 nm, respectively, pursuing the application simplicity.

A model to explain fluorescence as a function of riboflavin concentration was developed regressing reference values from those obtained by FFF (Equation (1) in Section 3.1).

### 2.2. Validation of the Prediction Model

For the validation, another independent set of samples was prepared by adding riboflavin concentrations of 0.0, 0.3, 0.7, 1.2, 1.8, 2.1, 2.4, and 2.7 ppm in reconstituted milk. As previously described, the FFF responses of milk samples were determined. Validation was performed by comparing the reference and the estimated concentration values (Equation 1) using linear regression. Further, a more robust prediction model for riboflavin concentration was developed regressing the reference values against those obtained by FFF with all data (calibration and validation sets), except for an outlier that was eliminated (Equation (2) in Section 3.2). This model was the one used for prediction of riboflavin concentration in commercial milks (Method 1 in Section 2.3).

### 2.3. Testing of the Model with Commercial Milks

Commercial reconstituted skim milk powder, and skim and whole ultra-high temperature (UHT) milk of several brands were analyzed for riboflavin concentration using the FFF method proposed and validated in the previous sections as well as by two HPLC reference methods.

The different commercial samples were split into three aliquots in order to determine riboflavin concentration by FFF (Method 1), the HPLC method of Albala-Hurtado et al. [3] (Method 2) as previously described, and at a certified milk laboratory using the HPLC method developed by Bueno-Solano et al. [1] (Method 3). Since HPLC methods required skimming of milk before quantification, for whole UHT milk samples, the FFF response (Method 1) was evaluated before (Method 1a) and after skimming (Method 1b) through centrifugation (4K-15, Sigma laboratory centrifuge, Osterode am Harz, Germany) at 12,000 × g during 20 min at 4 °C.

Data were processed by analysis of variance (ANOVA) using the general linear model (GLM) procedure of “Statistical Analysis System” (SAS, version 9.2, 2009, SAS Institute Inc., Cary, NC, USA). The least significant difference (LSD) test was used for comparison of sample data. Evaluations were based on a significance level of *p* < 0.05.

## 3. Results and Discussion

### 3.1. Calibration of the Prediction Model

**Figure 1:**
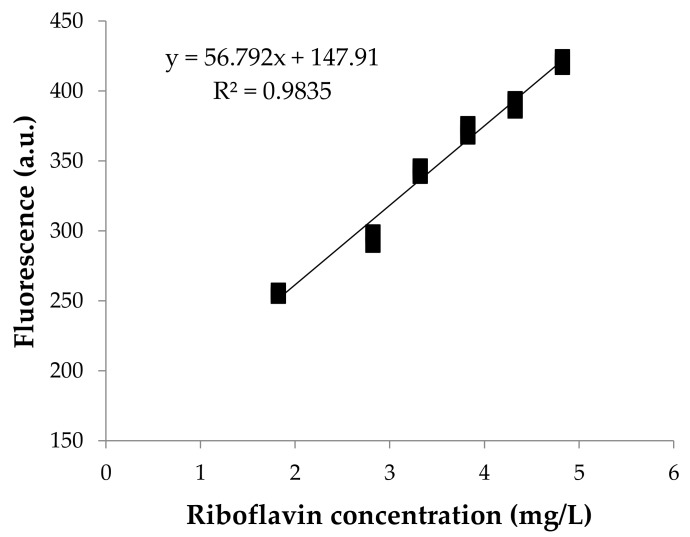
Fluorescence calibration curve at known concentrations of riboflavin in reconstituted skimmed milk powder. Three independent replications of each concentration are represented.

The relationship between the fluorescence response and the concentration of riboflavin is shown in Figure 1. FFF intensity and the concentration of riboflavin had a direct relationship, resulting in a linear calibration model with a coefficient of determination (R^2^) of 0.98, where the fluorescence of riboflavin at excitation and emission wavelengths of 370 and 530 nm, F, was related with total concentration of riboflavin (Rbf), following the next linear equation:

(1) $$F\;=\;56.79\cdot \left[\mathrm{Rbf}\right]\;+\;147.91.$$

It should be noticed that since Equation 1 has an intercept, its value of 147.91 a.u. would correspond to the fluorescence of milk with “theoretical absence of riboflavin”, which means that this fluorescence should be attributed to the matrix effect. However, apparently, this matrix effect would not depend on the cow skim milk type, as evidenced by the good results obtained when testing the model using commercial milks (Section 3.3.).

### 3.2. Validation of the Prediction Model

**Figure 2:**
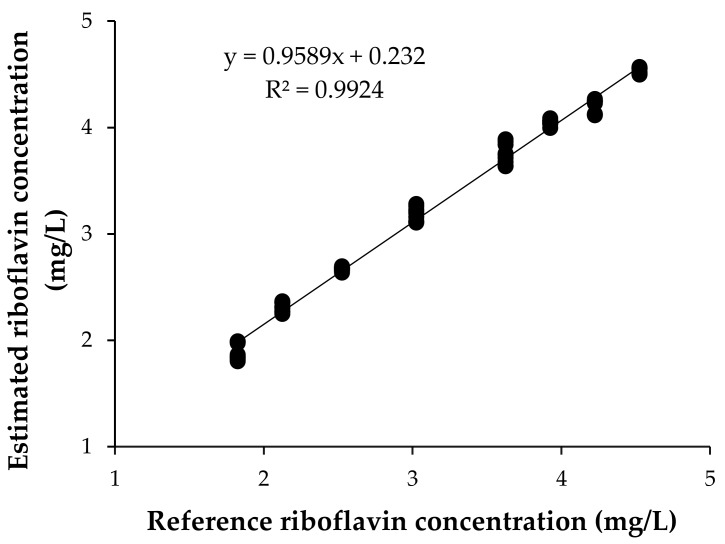
Relationship between estimated concentration of riboflavin and reference values. Three independent replications of each concentration are represented.

Figure 2 presents the riboflavin concentration of the samples corresponding to the validation set, predicted using the previously presented equation, versus their reference values. For validation, the correlation coefficient was R^2^ = 0.99, with a standard error of prediction (SEP) of 0.13 ppm and a coefficient of variation (CV) of 4.14% (*n* = 24). In addition, the student’s T-test, at a significance level of α = 0.05, indicated that there was a significant linear correlation between the estimated concentrations and reference values; therefore, the obtained model was successfully validated.

Once the method had been validated, it was decided to combine the calibration data set with the validation set to obtain a more robust model. In this process, an outlier data point was identified and eliminated from the subsequent analyses.

The model for predicting the concentration of riboflavin in milk obtained from the “extended calibration” set was

(2) $$\left[\mathrm{Rbf}\right]\;=\;\frac{F\;-\;160.4}{54.42}.$$

### 3.3. Testing of the Prediction Model with Commercial Milks

Table 1 shows the values of riboflavin present in the different samples of reconstituted commercial skimmed milk powder (SMP-1, SMP-2, SMP-3) and commercial skim UHT milk (sUHT-1, sUHT-2), all of them analyzed by the three different methods indicated in the Materials and Methods section (Method 1, FFF; Method 2, HPLC-UAB; and Method 3, HPLC-certified external laboratory). As it can be observed, the content of riboflavin found in milk samples (SMP-1, SMP-2, sUHT-1, and sUHT-2) was not significantly different (*p* > 0.05) when analyzed by the three methods, except in SMP-3 milk. In this case, results from Method 1 (FFF) were significantly different (*p* < 0.05) from the results from Methods 2 and 3 (HPLC methods). Since the only difference of SPM3 from the other two was that it was a product enriched with organic calcium and vitamin K, the enrichment affected the fluorescence response inducing the loss of validity of the calibration. It was also observed that the content of riboflavin in the SMP and UHT milk samples were slightly lower than those mentioned by Moreiras et al. [11], with average values of 2.04 and 1.7 mg/L, respectively. However, the results of the present study were within the range of other bibliographic references [12].

Commercial whole UHT milks analyzed without prior sample preparation, i.e., without centrifugation, showed a significant difference between the fluorescent method (Method 1a) and the HPLC methods, Methods 2 and 3 (Table 2). This was attributed to the high fat content of the samples, which increased the fluorescence intensity (note that the calibration was performed with skim milk powder). To avoid this interference, the samples were centrifuged before FFF quantification (Method 1b). After removing fat, no significant difference (*p* > 0.05) was observed between the different quantification methods.

The levels of riboflavin obtained in UHT whole milk in the present investigation were similar to those obtained by Amador-Espejo et al. [13]), Asadullah et al. [14], and Muñoz et al. [15], which ranged between 1.15 and 1.23 mg/L. However, Sunaric et al. [2] found values up to 1.81 mg/L, a value that is slightly higher than the obtained results. These differences in riboflavin concentration in milk may be due to the type of milk analyzed, influenced by factors such as breed, season, and feeding [16].

As the calibration was done by standard addition, the model is robust enough to deal with samples having a higher riboflavin concentration, as those reported by Moreiras et al. [11] or Sunaric et al. [2].

## 4. Conclusions

The results suggest the potential use of FFF as an immediate and simple method for the quantification of riboflavin in commercial skim milk. In whole milk, quantification with FFF implies a simple sample handling before fluorometric measurements, skimming. Since fluorescence was measured in arbitrary units, the method would require in-plant calibration in order to obtain the model coefficients, but using the same equation. Compared with conventional methods, it has the main advantage of being a fast, non-destructive technique. Besides, it does not require the use of reagents or qualified personnel, having potential for in-line measurements during processing. The method does not rely on emission spectra but on single determination of florescence intensity and does not require the application of chemometrics. To develop an industrial in-line sensor unit would require a relatively simple optoelectronics design, able to excite the sample at 370 nm and read FFF intensity at 530 nm through an adequate fiber optic probe. An in-line FFF sensor will be feasible as a process analytical technology (PAT) tool for industrial application in the dairy food sector and would be easy to adapt for immediate at- and off-line readings.

## Figures and Tables

**Table 1:** Quantification of riboflavin in reconstituted commercial skimmed milk powder (SMP) and skimmed UHT milk (sUHT) by different methods.

Methods	SMP-1	SMP-2	SMP-3	sUHT-1	sUHT-2
Method 1	1.69 ± 0.05 ^a^	1.70 ± 0.03 ^a^	1.84 ± 0.01 ^a^	1.22 ± 0.01 ^a^	1.26 ± 0.01 ^a^
Method 2	1.72 ± 0.01 ^a^	1.66 ± 0.02 ^a^	1.57 ± 0.03 ^b^	1.26 ± 0.01 ^a^	1.25 ± 0.05 ^a^
Method 3	1.82 ± 0.12 ^a^	1.62 ± 0.11 ^a^	1.54 ± 0.11 ^b^	1.30 ± 0.20 ^a^	1.30 ± 0.20 ^a^

Mean value ± standard deviation (SD) (mg/L). ^a, b^ Values per column without common superscripts were significantly different (*p* < 0.05); *n* = 9. SMP: skimmed milk powder; sUHT: skimmed ultra-high temperature milk. Method 1: Front-face fluorescence; Method 2: HPLC at UAB; Method 3: HPLC by certified external laboratory.

**Table 2:** Quantification of riboflavin in commercial whole UHT milks (wUHT) by different methods.

Methods	wUHT-1	wUHT-2
Method 1a	1.79 ± 0.06 ^a^	1.87 ± 0.10 ^a^
Method 1b	1.31 ± 0.03 ^b^	1.35 ± 0.06 ^b^
Method 2	1.29 ± 0.04 ^b^	1.24 ± 0.06 ^b^
Method 3	1.40 ± 0.20 ^b^	1.30 ± 0.20 ^b^

Mean value ± SD (mg/L). ^a, b^ Values per column without common superscripts were significantly different (*p* < 0.05); *n* = 12. wUHT: whole ultra-high temperature. Method 1a: direct front-face fluorescence (FFF); Method 1b: centrifugation and FFF; Method 2: HPLC at UAB; Method 3: HPLC by certified external laboratory.
